# Toll-Like Receptor Mediated Activation of Natural Autoantibody Producing B Cell Subpopulations in an Autoimmune Disease Model

**DOI:** 10.3390/ijms20246152

**Published:** 2019-12-06

**Authors:** Szabina Erdő-Bonyár, Judit Rapp, Tünde Minier, Gábor Ráth, József Najbauer, László Czirják, Péter Németh, Timea Berki, Diána Simon

**Affiliations:** 1 Department of Immunology and Biotechnology, Clinical Center, University of Pécs Medical School, H-7624 Pécs, Hungary; erdo-bonyar.szabina@pte.hu (S.E.-B.); rapp.judit@pte.hu (J.R.); najbauer.jozsef@pte.hu (J.N.); nemeth.peter@pte.hu (P.N.); simon.diana@pte.hu (D.S.); 2 Department of Rheumatology and Immunology, Clinical Center, University of Pécs Medical School, H-7632 Pécs, Hungary; minier.tunde@pte.hu (T.M.); czirjak.laszlo@pte.hu (L.C.); 3 Department of Pediatrics, Clinical Center, University of Pécs Medical School, H-7623 Pécs, Hungary; rath.gabor@pte.hu

**Keywords:** B cells, non-switched B cells, systemic sclerosis, dcSSc, TLR, CD180, RP105, CpG, IL-6, IL-10, natural autoantibodies, IgM, citrate synthase, DNA topoisomerase I

## Abstract

Altered expression and function of the Toll-like receptor (TLR) homologue CD180 molecule in B cells have been associated with autoimmune disorders. In this study, we report decreased expression of CD180 at protein and mRNA levels in peripheral blood B cells of diffuse cutaneous systemic sclerosis (dcSSc) patients. To analyze the effect of CD180 stimulation, together with CpG (TLR9 ligand) treatment, on the phenotype defined by CD19/CD27/IgD/CD24/CD38 staining, and function (CD69 and CD180 expression, cytokine and antibody secretion) of B cell subpopulations, we used tonsillar B cells. After stimulation, we found reduced expression of CD180 protein and mRNA in total B cells, and CD180 protein in B cell subpopulations. The frequency of CD180^+^ cells was the highest in the CD19^+^CD27^+^IgD^+^ non-switched (NS) B cell subset, and they showed the strongest activation after anti-CD180 stimulation. Furthermore, B cell activation via CD180 induced IL-6 and natural autoantibody secretion. Treatment with the combination of anti-CD180 antibody and CpG resulted in increased IL-6 and IL-10 secretion and natural autoantibody production of B cells. Our results support the role of CD180 in the induction of natural autoantibody production, possibly by NS B cells, and suggest an imbalance between the pathologic and natural autoantibody production in SSc patients.

## 1. Introduction

The production of scleroderma-specific autoantibodies and secretion of pro-inflammatory and pro-fibrotic cytokines by B cells is a well-described result that reflects immune dysregulation affecting B cells in systemic sclerosis (SSc) [1,2,3,4]. However, a large number of autoantibodies directed against well-conserved functional structures of the cell (e.g., nucleosome, DNA, nuclear and mitochondrial proteins, and receptors), the so-called natural autoantibodies that serve a protective function, can also be detected in healthy subjects and are dysregulated in patients with systemic autoimmune diseases [4,5].

Autoantibody production is a widely investigated function of B cells in SSc, but less attention has been devoted to their activation by innate immune receptors, including Toll-like receptors (TLRs), that are involved in recognizing pathogen- and damage-associated molecular patterns. CD180, or RP105 (radioprotective 105 kDa), is a TLR-like membrane protein that lacks an intracellular Toll-IL-1R (TIR) signaling domain [6]. CD180 was originally defined as a B cell surface molecule mediating polyclonal B cell activation, proliferation, and immunoglobulin production [7,8]. It was later described as a TLR homologue also expressed by monocytes and dendritic cells (antigen presenting cells), and the expression of CD180 correlated with TLR4 expression. CD180 and its helper molecule, MD-1, interact directly with the TLR4 signaling complex, inhibiting its ability to bind microbial ligands; thus, it serves as a negative regulator of TLR4 responses of antigen-presenting cells [6,9].

Differential expression and functions of CD180 on B cells have been associated with immune-mediated pathologies, including infection, chronic inflammation, and autoimmune disorders [6]. The severity of the disease in systemic lupus erythematosus (SLE) patients correlated with the amount of CD180-negative B cells in the peripheral blood [10,11]. CD180-negative peripheral blood B cells were also increased in patients with Sjögren’s syndrome; furthermore, these cells extensively infiltrated the salivary glands [12].

As the natural ligand of CD180 remains unknown, effects of crosslinking CD180 with monoclonal anti-CD180 antibody has been investigated. Anti-CD180 antibody activates over 85% of human and mouse B cells in vitro and induces robust immunoglobulin production [8]. The stimulation with anti-CD180 antibody synergizes with TLR9 ligands [8,13]. When CpG and anti-CD180 were used simultaneously, the proliferation of peripheral blood B cells was enhanced, and IgG and IgM production increased [13]. Simultaneous treatment with anti-CD180 antibody and LPS or CpG resulted in increased cytokine production of murine B cells [14].

In this study, we investigated the expression of CD180 at protein and mRNA levels in peripheral blood B cells of early diffuse cutaneous SSc (dcSSc) patients, and compared to healthy control (HC) B cells. We found that CD180 expression of dcSSc B cells was significantly lower than in HC B cells. To further investigate the role of CD180 in B cell activation, we used tonsillar B lymphocytes as a model. To investigate the CD180-mediated activation of B cell subsets, we used anti-CD180 antibody to ligate the receptor, and combined with treatment with CpG, a TLR9 ligand. Expression of CD180 in B cell subsets, and molecules of B cell activation, cytokine, and autoantibody production were analyzed. The frequency of CD180^+^ cells was the highest in non-switched memory (NS) B cells, which showed the strongest activation (CD69^+^) upon anti-CD180 stimulation. This activation was not influenced by the addition of CpG. Stimulation with anti-CD180 antibody alone, and in combination with CpG resulted in downregulation of CD180 protein and mRNA expression in total B cells, and decreased CD180 protein expression in B cell subsets. Activation via CD180 induced the elevation of IL-6 production and the anti-DNA topoisomerase I (anti-topo I) IgM natural autoantibody secretion, which were enhanced by the addition of CpG. Furthermore, treatment with the combination of anti-CD180 antibody and CpG resulted in increased IL-10 secretion and anti-citrate synthase IgM natural autoantibody production of B cells. Our results support the role of CD180 in the induction of natural autoantibody production, possibly by NS B cells, which are diminished in SSc patients, resulting in an imbalance between the pathologic and natural autoantibody production.

## 2. Results

### 2.1. CD180 Expression Is Decreased in dcSSc B Cells

**Figure 1:**
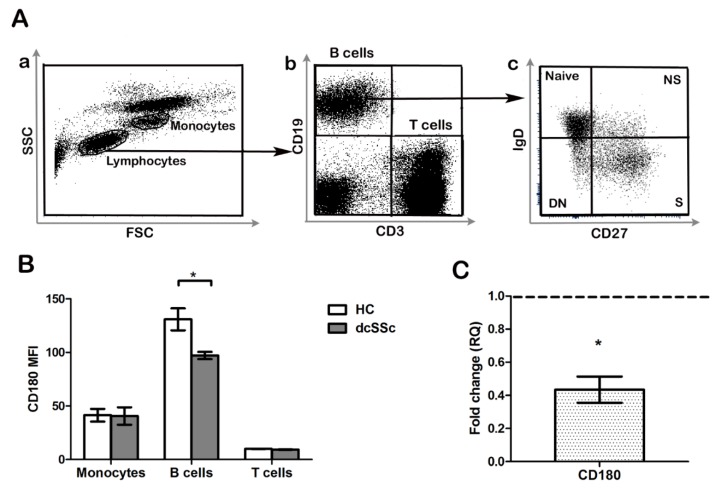
Analysis of CD180 expression in diffuse cutaneous systemic sclerosis (dcSSc) B cells. (**A**) Representative flow cytometry plots of peripheral blood leukocytes (**a**), B and T cells (**b**), and CD19^+^ B cells stained with CD27 and IgD defining the following four subsets: CD27^+^IgD^+^ non-switched memory (NS) B cells, CD27^+^IgD^−^ switched memory (S) B cells, CD27^−^IgD^+^ naive B cells, and CD27^−^IgD^−^ double negative (DN) B cells (**c**). (**B**) Flow cytometric analysis of CD180 expression in peripheral blood B cells, T cells, and monocytes of early untreated dcSSc patients compared to healthy controls (HCs). (**C**) CD180 mRNA expression in B cells of early untreated dcSSc patients compared to HCs. Gene expression was normalized to HCs and the horizontal line (value 1) represents the expression of control samples. Changes in gene expression are shown as relative quantification (RQ) values. Data are shown as mean ± standard error of the mean (SEM), *n* = 4 HC and *n* = 4 dcSSc, * *p* < 0.05.

Since it was described in other autoimmune diseases that differential expression of CD180 (RP105) might have a pathological role in B cell activation and autoantibody production [10,11,12,15], first we determined the CD180 expression of monocytes, T cells, and B cells. We compared the level (mean fluorescence intensity, MFI) of CD180 in PBMC samples of early, untreated dcSSc patients and HCs with flow cytometry. We found that the MFI of CD180 labeling was the highest in B cells, followed by monocytes, and T cells showed the lowest expression. The expression of CD180 in monocytes and T cells was similar in patients and HCs, while its level was significantly lower in B cells of dcSSc patients than in HC B cells (Figure 1A,B). Next, we examined the mRNA expression of the CD180 gene in purified B cells of early, untreated dcSSc patients and compared to HCs. We found highly downregulated expression of CD180 mRNA in dcSSc patients (Figure 1C).

### 2.2. TLR Ligation Results in Reduced CD180 mRNA and Protein Expression of B Cells

**Figure 2:**
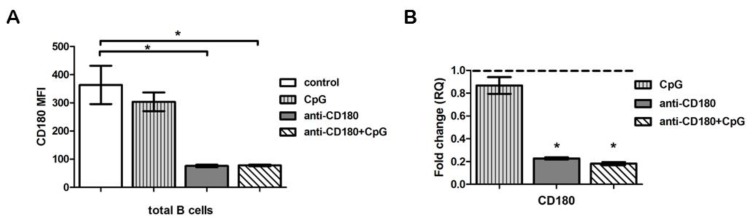
Effect of Toll-like receptor (TLR) stimulation on CD180 protein and mRNA expression. (**A**) CD180 expression of unstimulated (control), CpG, anti-CD180 antibody-stimulated, and anti-CD180 + CpG-treated (24 h) tonsillar B cells (mean fluorescence intensity, MFI). (**B**) CD180 mRNA expression in tonsillar B cells following CpG, anti-CD180, and anti-CD180 + CpG stimulation (24 h). Changes in gene expression are shown as RQ values, normalized to unstimulated controls. The horizontal line (value 1) represents the CD180 mRNA of unstimulated control samples. Data are shown as mean ± SEM, *n* = 4, * *p* < 0.05.

The CD180-negative B cells were described as highly activated cells in SLE [11], and stimulation via CD180 is known to activate B cells [6]. Furthermore, TLR ligands were reported to downregulate the mRNA expression of CD180 molecule [16], thus we hypothesized that the decreased CD180 expression of dcSSc B cells could be a result of activation through TLRs. To investigate whether TLR stimulation leads to diminished expression of CD180 molecules in B cells, we stimulated tonsillar B cells with anti-CD180 antibody. We measured the expression of CD180 at protein and mRNA levels, and found that following anti-CD180 ligation, the MFI and mRNA levels of CD180 significantly decreased (Figure 2A,B). To study the influence of other TLR ligands on the activation via CD180, we co-treated the B cells with CpG, and found that the expression of CD180 was similar to anti-CD180-stimulated cells both at protein (Figure 2A) and mRNA (Figure 2B) levels. Treatment with CpG alone did not result in changes of CD180 MFI (Figure 2A) or CD180 mRNA (Figure 2B) levels in B cells.

### 2.3. The Frequency of CD180^+^ Cells Is the Highest in the Non-Switched Memory B Cell Subset

**Figure 3:**
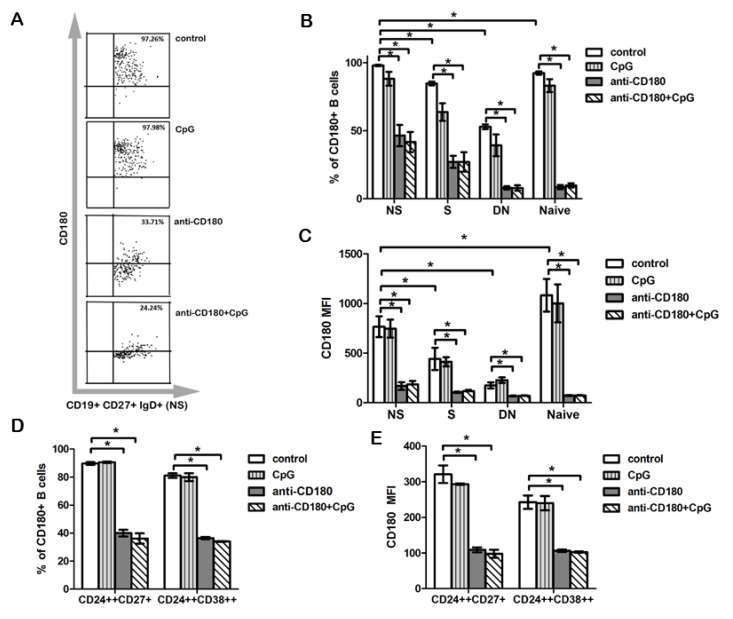
Effect of TLR stimulation on CD180 expression in B cell subpopulations. Flow cytometric analysis of the distribution of CD180^+^ B cells and CD180 MFI in CD27^+^IgD^+^ non-switched memory (NS), CD27^+^IgD^−^ switched memory (S), CD27^−^IgD^+^ naive, and CD27^−^IgD^−^ double negative (DN) B cell subsets (**A**–**C**), and in CD24^high^CD38^high^ and CD24^high^CD27^+^ Breg subsets (**D,E**). Representative dot plots show the changes of CD180 positivity in control and treated NS B cells (**A**). Changes of CD180^+^ cell ratios (**B**) and CD180 MFI (**C**) in different B cell subsets defined by CD27 and IgD staining after CpG, anti-CD180 antibody stimulation or anti-CD180 + CpG treatment. Changes of CD180^+^ cell ratios (**D**) and CD180 MFI (**E**) in Breg subsets defined by CD24, CD27, and CD38 staining after CpG, anti-CD180 antibody stimulation or anti-CD180 + CpG treatment. Data are presented as means ± SEM, *n* = 4, * *p* < 0.05.

To assess phenotypical and functional alterations of B cells upon anti-CD180 stimulation, first we investigated the expression of CD180 in B cell subsets, defined by CD27 and IgD labeling (Figure 1A). Using tonsillar B cells, we analyzed the following subpopulations: CD27^+^IgD^+^ non-switched memory (NS) B cells, CD27^+^IgD^−^ switched memory (S) B cells, CD27^−^IgD^+^ naive B cells (N), and CD27^−^IgD^−^ double negative (DN) B cells. We found that the percentage of CD180^+^ cells was significantly higher in NS B cells compared to all other subsets, namely, naive, S, and DN B cells (Figure 3A,B). Next, we measured the changes in the percentage of CD180^+^ B cells in the NS, S, naive, and DN B cell subpopulations upon anti-CD180 stimulation, and found that the frequency of CD180^+^ cells was significantly decreased in all four B cell subsets (Figure 3B). Addition of CpG to the anti-CD180 antibody-treated B cells did not result in further changes in the ratio of CD180^+^ B cell subpopulations (Figure 3B). Treatment with CpG alone did not reduce the percentage of CD180^+^ cells in the investigated B cell subsets (Figure 3B). The overall pattern of the changes in CD180 MFI in the investigated B cell subsets was similar to that found in the frequency of CD180^+^ cells, but the CD180 MFI in unstimulated B cells was the highest in naive B cells (Figure 3C). We also investigated the expression of CD180 in regulatory B cells (Bregs). There is still no consensus on the phenotype of Bregs, multiple subsets with many similarities in phenotype and effector functions have been described [17]. In humans, both CD19^+^CD24^high^CD38^high^ [18] and CD19^+^CD24^high^CD27^+^ [19] Bregs have been defined. Based on these findings, we analyzed the CD180 expression of Breg subsets with these phenotypes using flow cytometry. We found that the percentage of CD180+ cells and the MFI of CD180 labeling was significantly decreased after anti-CD180 antibody treatment, and also after combined treatment with anti-CD180 antibody and CpG (Figure 3D,E).

### 2.4. Anti-CD180 Stimulation Resulted in Activation of B Cell Subsets

**Figure 4:**
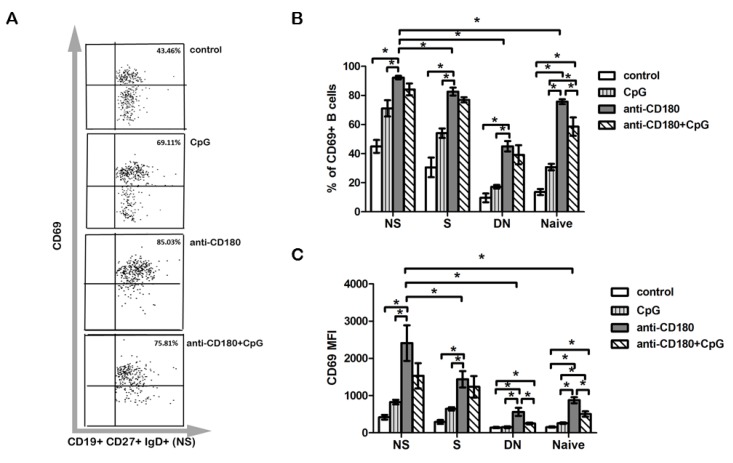
Expression of CD69 activation marker on B cell subpopulations. Representative dot plots show the changes of CD69 positivity in control and treated NS B cells (**A**). The percentage of CD69^+^ (**B**) B cells and CD69 MFI (**C**) in CD27^+^IgD^+^ non-switched memory (NS), CD27^+^IgD^−^ switched memory (S), CD27^−^IgD^+^ naive, and CD27^−^IgD^−^ double negative (DN) B cell subsets defined by CD27 and IgD in anti-CD180 antibody, CpG-stimulated, and in anti-CD180 and CpG co-treated tonsillar B cells. Data are presented as means ± SEM, *n* = 4, * *p* < 0.05.

We also examined the activation of the B cell subsets defined by CD27 and IgD labeling after 24 h of anti-CD180, CpG, and anti-CD180 + CpG stimulation by detecting CD69, an early activation marker. Upon anti-CD180 stimulation, the frequency of CD69^+^ cells was increased in all investigated subsets compared to CpG stimulation, showing the highest ratio in NS B cells (Figure 4A,B). This result is consistent with our observation that NS B cells show the highest ratio of CD180^+^ cells. CpG, when used together with anti-CD180 antibody stimulation, did not alter the percentage of CD69^+^ cells in NS, S, or DN B cells compared to anti-CD180 stimulation alone. However, a significant decrease in the frequency of CD69^+^ naive B cells was observed, compared to anti-CD180 ligation alone (Figure 4B). The overall pattern of the changes in CD69 MFI was similar to that found in the frequency of CD69^+^ cells, and after anti-CD180 antibody stimulation, the CD69 MFI was the highest in NS B cells. Yet, CD69 MFI was not significantly different between CpG- and anti-CD180 + CpG-stimulated DN B cells, while combined anti-CD180 and CpG treatment compared to anti-CD180 antibody stimulation alone significantly reduced the MFI of CD69 in DN B cells (Figure 4C).

### 2.5. TLR Stimulation Differentially Affects IL-6 and IL-10 Production of B Cells

**Figure 5:**
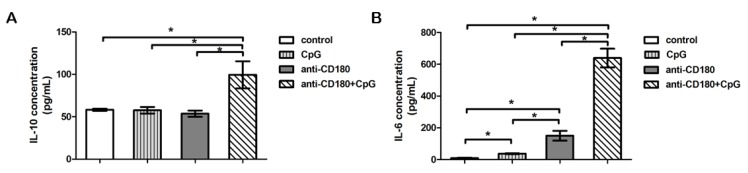
TLR induced cytokine production of total B cells. Detection of IL-6 (**A**) and IL-10 cytokines (**B**) in the supernatant of tonsillar B cells stimulated with CpG, anti-CD180 antibody, or anti-CD180 + CpG, or left unstimulated (control), as measured by ELISA. Data are presented as means ± SEM, *n* = 4, * *p* < 0.05.

Recently, it was described that prominent IL-6 production by activated B cells promotes spontaneous germinal center formation and plasma cell differentiation, further supporting the role of this cytokine in the pathogenesis of systemic autoimmune diseases [20]. To test whether anti-CD180 stimulation influences B cell–derived IL-6 production, or induces the production of Breg cytokine IL-10, we measured the concentration of these cytokines in the supernatant of separated B cells after 24 h of stimulation. Anti-CD180 stimulation alone significantly increased the concentration of IL-6 in the supernatant of tonsillar B cells compared to both unstimulated and CpG-stimulated cells, while it had no significant effect on the production of IL-10. CpG on its own significantly elevated the concentration of IL-6 in the supernatant, but did not increase the production of IL-10. However, supplementing anti-CD180 antibody with CpG significantly augmented the production of both IL-6 and IL-10 compared to unstimulated, CpG-, and anti-CD180-stimulated B cells (Figure 5A,B).

### 2.6. CD180 Stimulation Induces Natural Autoantibody Production

**Figure 6:**
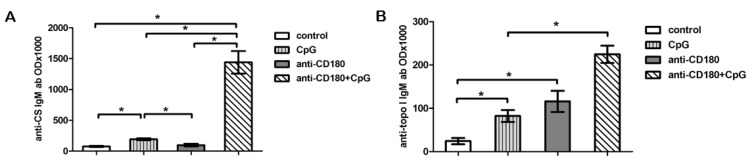
Induction of natural autoantibody production by TLR stimulation. Anti-citrate synthase IgM and (**A**) anti-DNA topoisomerase I IgM (**B**) production of B cells stimulated with CpG, anti-CD180 antibody, or anti-CD180 + CpG, or left unstimulated (control). Data are presented as means ± SEM, *n* = 3, * *p* < 0.05.

Since CD180 stimulation resulted in the most pronounced activation of NS memory B cells, resembling the B1 B cell population, which includes cells responsible for natural IgM autoantibody production [21], we investigated the effect of CD180 ligation on the production of natural autoantibodies by tonsillar B cells. In our previous studies, we detected anti-citrate synthase (CS) IgM antibodies in HCs and patients with autoimmune diseases [22,23]. Here we measured the anti-CS IgM autoantibody in the supernatant of anti-CD180-stimulated and control tonsillar B cells after 7 days of culture, but did not find any differences between the control and anti-CD180-stimulated samples. Treatment with CpG alone resulted in a significant increase in the level of anti-CS IgM in the supernatant. Combined treatment with anti-CD180 antibody and CpG significantly increased the production of anti-CS IgM autoantibody compared to unstimulated, CpG-, and anti-CD180-stimulated B cells (Figure 6A).

Previously, we reported that different epitopes of topoisomerase I (Scl-70) induce not only pathologic autoantibodies in dcSSc patients, but the antigen has epitopes (including F4) that induce natural autoantibody production in healthy individuals as well [24]. Therefore, we also measured the level of anti-topoisomerase I F4 fragment (anti-topo I) antibody in the supernatant of CD180-stimulated B cells. Anti-CD180 itself significantly raised the level of anti-topo I IgM antibodies in the supernatant of tonsillar B cells similarly to CpG, and the level was further elevated with addition of CpG (Figure 6B). Further, we measured the production of pathologic autoantibodies in the supernatant of anti-CD180- and anti-CD180 + CpG-stimulated tonsillar B cells. Antinuclear antibodies (ANA), anti-dsDNA, and anti-nucleosome antibodies were not detectable in the supernatant of B cells under the investigated conditions.

## 3. Discussion

In SSc, data on the role of innate immune molecules in the dysregulated B cell functions are scarce. The TLR homologue CD180 molecule activates the majority of B cells, resulting in phenotypic and functional alterations [8,14,25]. CD180-negative B cells, macrophages, and dendritic cells were shown to be elevated in SLE and in lupus-prone mice [15,26]. Here, we demonstrated that the expression of CD180 is decreased in early dcSSc peripheral blood B cells compared to HCs, while the percentage of CD180-negative monocytes was unaltered, emphasizing the potential role of CD180 in B cell dysfunction in SSc. CD180-negative B cells in SLE were described as highly activated cells [15], and CD180 can be internalized after ligation by anti-CD180 antibody [14]; thus, B cell activation via CD180 might be a possible explanation for the decreased expression of CD180 in SSc B cells.

We were the first to investigate the distribution of CD180 molecules in tonsillar B cell subpopulations defined by CD27 and IgD labeling, and found that the frequency of CD180^+^ cells was the highest among NS cells. The percentage of CD180^+^ cells was significantly decreased in all subsets upon anti-CD180 stimulation, suggesting that ligation of CD180 triggered its internalization. Interestingly, we also found that anti-CD180 stimulation downregulated the mRNA expression of CD180, strengthening the possibility of autoregulation of CD180 expression by B cells. Signaling via TLRs can interfere with CD180 signals, since ligands of TLR7 and TLR9 have already been shown to downregulate CD180 expression in human peripheral blood B cells [16]. Nevertheless, according to our results, co-treatment with anti-CD180 and CpG reduced the expression of CD180 to a similar level as anti-CD180 did on its own, while CpG treatment alone had no significant effect on the expression of CD180. All investigated B cell subsets were activated by anti-CD180 and anti-CD180 and CpG co-treatment, as indicated by CD69 expression.

Anti-CD180 antibody is known to activate marginal zone (MZ) B cells and induces overexpression of CD69 in mice [14]. NS B cells in human peripheral blood represent MZ-derived B cells [21], and according to our results, ratio of CD69^+^ cells was the highest among NS B cells upon anti-CD180 stimulation. Moreover, the combination of anti-CD180 stimulation with CpG showed no additional effect on the activation of NS B cells.

We have already described that the percentage of NS B cells is lower in SSc than in HC [27]. NS B cells resemble B1 B cells with innate-like features [21], suggesting that they produce natural autoantibodies. Natural IgM autoantibodies are polyreactive and serve as scavengers of damaged molecules and cells, and therefore have been implicated in the control of inflammation and autoimmune diseases [28]. The therapeutic effects of intravenous immunoglobulins (IVIg) could partly be due to the appropriate pool of natural autoantibodies [29], and IVIg seems to be beneficial in SSc [30]; therefore, we also examined the effect of anti-CD180 and anti-CD180 and CpG co-treatment on the production of natural and pathologic autoantibodies of tonsillar B cells. Pathologic autoantibodies were undetectable in the supernatant of cells, while anti-CD180 and CpG co-treatment significantly enhanced the production of both anti-CS and anti-topoisomerase I IgM antibodies, suggesting the synergistic beneficial effect of anti-CD180 antibody and TLR9 ligand on natural antibody production of B cells. Interestingly, anti-CD180 stimulation increased the level of anti-topoisomerase I IgM, while it had no influence on anti-CS IgM production. However, CpG stimulation increased the level of anti-topoisomerase I IgM and anti-CS IgM as well. CS molecule is not the target of a disease-specific pathologic antibody, while natural anti-topoisomerase I autoantibodies recognize distinct epitopes of topoisomerase I, which is the target antigen of an SSc-specific pathologic autoantibody (anti-Scl-70) [24]. The onset of autoimmune diseases may correlate with a switch from production of self-reactive low-affinity IgM to high-affinity IgG isotype autoantibodies by B cells. B cell activation via CD180 may have a role in regulating the level of natural IgM antibodies directed against the target antigens of pathologic antibodies.

The serum concentration of IL-6 is elevated both in SSc [31] and SLE patients [32]; furthermore, IL-6 production by B cells drives autoimmune germinal center formation in a mouse model of SLE, promoting the disease [20]. We found that ani-CD180 stimulation enhanced the IL-6 production by tonsillar B cells, which was further augmented by the addition of CpG. IL-6 was shown to have a pronounced effect on plasma cell survival [33], and if combined with IL-2 or IL-10, IL-6 enhanced the generation of early plasma cells [34]. Moreover, in SLE patients, endogenous IL-6 produced by B cells bound to IL-6R of B cells and drove them into terminal differentiation [35]. Consequently, B cell activation via CD180 alone or together with the TLR9 ligand may help plasma cell differentiation and antibody production. Bregs are decreased and functionally impaired in SSc patients [36], thus we also investigated the effect of anti-CD180 treatment on the IL-10 production of B cells. We found that CpG alone did not induce the production of IL-10, which is in agreement with the findings of Gantner et al. [37]. The concentration of IL-10 was increased only when anti-CD180 antibody was combined with CpG, suggesting their synergisitc effect on Bregs. However, CD180 expression was significantly decreased in CD19^+^CD24^high^CD38^high^ and CD19^+^CD24^high^CD27^+^ Breg subsets after anti-CD180 antibody treatment, and also after combined treatment with anti-CD180 antibody and CpG, suggesting that anti-CD180 antibody stimulation alone may influence Breg functions other than the production of IL-10.

Our findings suggest that the TLR homologue CD180 molecule may be involved in B cell dysfunction in early dcSSc. We also described the effects of anti-CD180 activation on natural autoantibody and cytokine production of tonsillar B cells and conclude that our data raise the possibility that anti-CD180 stimulation might shift the type of antibodies produced by B cells towards the natural autoantibodies.

## 4. Materials and Methods

### 4.1. Patients

Diffuse cutaneous systemic sclerosis patients (dcSSc) enrolled for the gene expression and flow cytometric studies all fulfilled the 2013 ACR/EULAR SSc classification criteria. Disease duration was 1.75 ± 0.96 years, age at enrollment was 39 ± 22.73 years. None of the patients were on current or previous immunosuppressive therapy. The controls were age- and sex-matched healthy individuals (HC). All participants gave their informed consent to the study following approval by the Hungarian National Ethics Committee (ETT TUKEB 47861-6/2018/EKU) and Regional Clinical Research Ethics Committee (7724-PTE 2019).

### 4.2. Mononuclear Cell Isolation, B Cell Separation

Peripheral blood mononuclear cells (PBMCs) were isolated by Ficoll-Paque Plus (GE Healthcare, Chicago, IL, USA) density gradient centrifugation of peripheral blood samples (SSc *n* = 4, HC *n* = 4). Negative selection of B cells was performed using the MACS B cell isolation kit II (Miltenyi Biotech, Bergisch Gladbach, Germany), according to the manufacturer’s protocol. B cell purity was >95%.

Tonsil tissue was collected from asymptomatic children who underwent routine tonsillectomy at the Department of Pediatrics, University of Pécs. After surgical removal, the tonsils were transported immediately to the laboratory and they were prepared the same day.

Tonsils were manually homogenized, and the cell suspension was filtered through a 70 µm sterile cell strainer, followed by isolation of mononuclear cells and separation of B cells, as described above.

### 4.3. Cell Stimulation

For RNA isolation, B cell subset analysis and cytokine measurements, 3 × 10^5^ B cells were stimulated with LEAF Purified anti-human CD180 (RP105) antibody (Clone: MHR73-11) (BioLegend, San Diego, CA, USA) at 1 µg/mL (hereafter referred to as anti-CD180) or anti-CD180 in combination with 1 µg/mL CpG (Hu/Ms CpG-B DNA, ODN2006, Hycult Biotech, Wayne, NJ, USA) or with CpG alone or were left unstimulated for 24 h at 37 °C. To assess natural and pathologic antibody production, 4 × 10^5^ B cells were stimulated with anti-CD180, or CpG or anti-CD180 in combination with CpG, or left unstimulated for 7 days at 37 °C.

### 4.4. RNA Isolation, cDNA Synthesis, and qPCR

Total RNA was extracted from isolated B cells using the NucleoSpin RNA XS kit (Macherey-Nagel Inc, Bethlehem, PA, USA). Following cDNA generation (High Capacity cDNA Reverse Transcription Kit, Thermo Fisher Scientific, Waltham, MA, USA), the CD180 mRNA expression was analyzed individually in dcSSc patients (*n* = 4) and HCs (*n* = 4) using Applied Biosystems TaqMan Gene Expression Assays (Thermo Fisher Scientific, Waltham, MA, USA). To determine the CD180 mRNA expression of tonsillar B cells (*n* = 4), the SensiFAST SYBR Lo-ROX Kit (Bioline, London, UK) was used. Amplifications were performed using an Applied Biosystems 7500 RT-PCR System (Thermo Fisher Scientific, Waltham, MA, USA). Gene expression was analyzed with 7500 Software v2.0.6 (Thermo Fisher Scientific, Waltham, MA, USA) and normalized to GAPDH (a “housekeeping” gene) as reference. Fold changes (RQ) were calculated according to the 2-ddC_T_ method.

### 4.5. Flow Cytometric Analysis

To measure the expression of CD180 of B cells, PBMCs from dcSSc patients (*n* = 4) and HCs (*n* = 4) were labeled with the combination of anti-human CD19-AmCyan (SJ25C1, Becton Dickinson, Franklin Lakes, NJ, USA) and anti-human CD180-PE (G28-8, Becton Dickinson, Franklin Lakes, NJ, USA) antibodies. Multiparametric flow cytometry was performed on tonsillar B cells (*n* = 4) with antibodies specific for CD19, CD27, IgD, CD24, CD38, CD180, and CD69. Purity of B cells was assessed using anti-human CD19-AmCyan antibody. To distinguish between memory B cell subsets and to evaluate the expression of CD180 and the activation marker CD69, four-color analysis was conducted using the combination of anti-human CD27-PE/Cy7 (M-T271, BioLegend, San Diego, CA, USA), anti-human IgD-PerCP (IA6-2, BioLegend, San Diego, CA, USA), anti-CD180-PE (G28-8, Becton Dickinson, Franklin Lakes, NJ, USA), and anti-human CD69-APC/Cy7 (FN50, BioLegend, San Diego, CA, USA), while Breg subsets were defined by anti-human CD27-PE/Cy7, anti-human CD24-BV421 (ML5, BioLegend, San Diego, CA, USA), and anti-human CD38-APC/Cy7 (HIT2, BioLegend, San Diego, CA, USA) antibodies following the manufacturer’s instructions. Briefly, PBMCs or separated B cells were incubated with the appropriate antibodies for 30 min on ice, washed twice in phosphate-buffered saline (PBS), and fixed with FACSFix (0.5% PFA in PBS). Fluorescence of the labeled cells was recorded using a FACS Canto II flow cytometer (Becton Dickinson, Franklin Lakes, NJ, USA) and analyzed with FCS Express 6 software (De Novo Software, Pasadena, CA, USA).

### 4.6. IL-10 and IL-6 ELISA

Supernatant of anti-CD180, CpG, and CpG + anti-CD180 stimulated, and unstimulated B cells were collected (*n* = 4) and stored at −80 °C until being measured. IL-10 and IL-6 production was quantified using Human IL-10 and IL-6 DuoSet ELISA kits (Bio-Techne, Minneapolis, MN, USA) according to the manufacturer’s protocols. The reaction was developed with TMB and measured at 450 nm using an iEMS MF microphotometer (Thermo Labsystem, Beverly, MA, USA).

### 4.7. Measurement of Natural and Pathologic Autoantibodies

The supernatant obtained from tonsillar B cells treated with anti-CD180, CpG, or CpG + anti-CD180, or left unstimulated were collected (*n* = 3) and stored at −80 °C until being measured. The levels of anti-citrate synthase (anti-CS) IgM autoantibodies and anti-topoisomerase I (fragment F4) IgM autoantibodies were determined with in-house ELISAs, as previously described [23,24]. The amounts of autoantibodies against dsDNA, nucleosome, and antinuclear antibodies (ANA) in the supernatant of tonsillar B cells were measured using commercial ELISA kits (ORGENTEC Diagnostika GmbH, Mainz, Germany). The reaction was developed with TMB and measured at 450 nm using an iEMS MF microphotometer (Thermo Labsystem, Beverly, MA, USA).

### 4.8. Statistical Analysis

Statistical evaluation was performed with SPSS v. 25.0 statistics package (IBM, Armonk, NY, USA) using Student’s *t*-tests and ANOVA where *p*-values < 0.05 were considered significant.

## Abbreviations

ANA	Antinuclear antibodies
Breg	Regulatory B cell
CpG	5’—C—phosphate—G—3’ dinucleotide
CS	Citrate synthase
dcSSc	Diffuse cutaneous systemic sclerosis
DN B	Double negative B cell
ELISA	Enzyme-linked immunosorbent assay
FSC	Forward scatter
GAPDH	Glyceraldehyde 3-phosphate dehydrogenase
HC	Healthy control
Hu	Human
LEAF	Low endotoxin, azide-free
MFI	Mean fluorescence intensity
Ms	Mouse
MZ	Marginal zone
NS B	Non-switched B cell
PBMC	Peripheral blood mononuclear cells
PFA	Paraformaldehyde
qPCR	Quantitative polymerase chain reaction
RQ	Relative quantification
S B	Switched B cell
SLE	Systemic lupus erythematosus
SSc	Systemic sclerosis
SSC	Side scatter
TLR	Toll-like receptor
TMB	3,3′,5,5′-Tetramethylbenzidine
Topo I	Topoisomerase I
